# Are serum eosinophilic cationic protein levels of toll collectors affected by diesel exhaust exposure?

**DOI:** 10.12669/pjms.325.10844

**Published:** 2016

**Authors:** Cahit Bilgin, Peri Arbak, Ozlem Yavuz, Ege Gulec Balbay, Oner Balbay, Ali Nihat Annakkaya

**Affiliations:** 1 Dr. Cahit Bilgin, MD. Assistant Professor, Department of Chest Diseases, Sakarya University, Medical School, Sakarya, Turkey; 2 Prof. Dr. Peri Arbak, MD. Department of Chest Diseases, Duzce University, Medical School, Duzce, Turkey; 3 Prof. Dr. Ozlem Yavuz, MD. Department of Medical Biochemistry, Balikesir University, Medical School, Balikesir, Turkey; 4 Dr. Ege Gulec Balbay, MD. Associate Professor, Department of Chest Diseases, Duzce University, Medical School, Duzce, Turkey; 5 Prof. Dr. Oner Balbay, MD. Department of Chest Diseases, Duzce University, Medical School, Duzce, Turkey; 6 Prof. Dr. Ali Nihat Annakkaya, MD. Department of Chest Diseases, Duzce University, Medical School, Duzce, Turkey

**Keywords:** Diesel exhaust, Pulmonary function, PEF variability, ECP, Toll collectors

## Abstract

**Objective:**

There are few studies on the diesel exhaust particulates (DEP) / eosinophilic cationic protein (ECP) level relationship. This study aimed to detect ECP levels in a highly DE exposed group, named as toll collectors.

**Methods:**

In a cross-sectional study, levels of serum ECP, rates of respiratory symptoms, mean levels of respiratory functions, smoking status, and variations in peak expiratory flow (PEF) during weekends and working days were compared for 68 toll collectors (TC) (range of age, 24-48 years) and 28 controls (range of age, 25-61 years). All subjects in the study group were men.

**Results:**

No significant difference was observed in terms of symptoms and smoking rates between the toll collectors and control group. The number of toll collectors [12/68 (17.7%) vs 1/28 (3.5%)] with diurnal PEF variability in the working period was higher than that of controls (p=0.058). Mean ECP level of toll collectors was higher than that of controls (32.8 vs 21.4 ng/L), but the difference was not significant. Mean ECP levels were higher in subjects experiencing diurnal PEF variability during work and off-work periods (34.9 vs 28.3 ng/L, p=0.410).

**Conclusions:**

Serial PEF measurements combined with serum ECP measurements did not add a new tool to detect the sensitivity of workers dealing with DE. Much more diesel exhaust exposed workers should be included to search for cheap and available methods when evaluating airway.

## INTRODUCTION

There is increasing concern about the possible adverse effects of diesel exhaust particulates (DEP) on human health.^1^ People working around diesel equipment or living close to major roadways are more likely to present irritant respiratory symptoms and reduced baseline pulmonary function,^2^ as well as develop respiratory diseases such as allergy,^3^ chronic obstructive pulmonary disease,^4^ and lung cancer.^5^ Previous studies had shown an association between chronic exposure to automobile exhaust and adverse effects on respiratory symptoms and pulmonary function in humans.^6^ It had also been reported that asthma could develop due to the exposure of excessive diesel exhaust, while exacerbations might be seen in asthmatic patients exposed to diesel particulates.^1^ Among inflammation markers of asthma, Eosinophilic Cationic Protein (ECP) levels reflect the intensity of eosinophilic airway inflammation, as well as the disease activity. Serum levels of ECP have been shown to correlate with the severity of bronchial asthma.^7^ Noah et al. demonstrated a significant increase in ECP levels of upper airway secretions following exposure to diesel particulates.^8^ The ECP levels in sputum of asthmatic patients after diesel particles exposure were found high in another study.^9^ A comparative study showed that ECP levels in bronchial washing of mild asthmatics were higher than those of healthy subjects after DE exposure.^10^

A few studies which focused on manual tollbooth collectors have reported acute irritative symptoms due to nasal and throat inflammation, nausea, headache, and lower peak expiratory flow rates.^11^,^12^ Epidemiological studies have demonstrated an association between DE and respiratory diseases, but the underlying mechanisms are still not fully understood, and exposure must to be better characterized to improve the basis for risk assessment in occupational and environmental settings.^13^

Although ECP has been studied, less, we thought that chronic exposure to diesel might lead an eosinophilic airway inflammation and result an increase in serum ECP levels. To our knowledge, this is the first study that carries out a search to detect ECP levels in a highly DE exposed group, named as toll collectors.

## METHODS

In a cross-sectional study the levels of serum ECP, respiratory symptoms and smoking status of 68 toll collectors (TC) (mean age, 37.5 ± 6.8 years) and 28 control cases (mean age, 38.1 ± 8.9 years) were randomly selected among completely healthy, age-matched hospital personnel and compared. The toll collectors were employed in manual tollbooths in the Kaynasli, Golyaka-Duzce region, a city in the northwest part of Turkey. The study group was located on the busiest line of the trans-European motorway. Employees worked an average of 8 hour a day in tollbooths. The number of toll collectors working in manual tollbooths in the Kaynasli, Golyaka-Duzce region was 120. Exclusion criteria were subjects with ischemic heart disease and chronic obstructive lung disease and body mass index over 30. Hence, 68 out of 120 toll collectors accepted to participate into the study.

The mean levels of forced vital capacity (FVC) predicted %; forced expiratory flow in first Second (FEV_1_) predicted %; FEV_1_/FVC, Maximal Midexpiratory Flow Rate (MMEF) predicted %; and the diurnal variabilities of Peak Expiratory Flow (PEF) rates in weekends and working days were compared. All of the study subjects and controls were men. The subjects in both groups had body mass index values under 30. All examinations and tests were carried out at Duzce University Hospital.

A respiratory questionnaire concerning respiratory complaints (dyspnea, cough, sputum and chest tightness), previous pulmonary diseases, family history for respiratory diseases, and smoking status was completed by all subjects.^14^ Questionnaires were administered by the same physician who performed the physical examination and spirometric measurements. The original European Community for Coal and Steel (ECSC) Questionnaire on Respiratory Symptoms was translated from English into Turkish and then back to English.

Serum ECP levels were measured by chemiluminescence method in the Immulite One analyzer (Immulite One ECP, DPC Los Angeles, CA, USA). The detection limit and normal values of immunometric ECP assay were 0.2 ng/mL and 0-24 ng/mL, respectively. An informed consent was obtained from all subjects, and the study was approved by the Duzce University Faculty of Medicine Ethics Committee.

Spirometric measurements were performed using the same spirometer (Vitalograph Alpha). All measurements were carried out in February & March, 2007. Calibrations and the measurements were performed in accordance with guidelines recommended by American Thoracic Society (ATS).^15^ According to the ATS criteria, individual spirograms were acceptable if exhalation time was satisfactory (e.g. 6 sec). Measurements fulfilled the reproducibility criteria if the two largest FEV1 and FVC were within 200 ml of each other among three acceptable spirograms. Recorded variables were FVC and FEV1, as well as maximal expiratory flow at 25–75% of FVC (MMF). The lung function variables were expressed in terms of absolute values and as percentages of the predicted values.

A Mini-Wright peak flowmeter and diary card were used for PEF monitoring. First, subjects were trained in the use of the peak flowmeter and how to record the measurements of PEF on working days and record exposure on a diary card. Toll collectors and controls also were told to record their PEF four times daily (upon waking, noon, after work, and before bedtime) for at least one week to 10 days when away from work. All subjects recorded their PEF measurements for one month. The sum of the weekend records at least one week to 10 days was accepted as PEF monitoring away from work, while the remaining PEF records reflected PEF monitoring at working period. For analyzing PEF records, a quantitative method was used.^16^ The following criteria were used: (1) a 20% or greater diurnal variability of peak flow readings to make a diagnosis of asthma; (2) occurrence of such changes relatively more frequently on working days than off-work days; and (3) exclusion of indeterminate findings such as occurrence of diurnal change only on one occasion or occurrence of PEF changes without diurnal basis.

The χ^2^ test was used for testing differences in the prevalence of respiratory symptoms among the groups. A comparison of spirometric measurements and serum ECP levels were performed using a t-test for two independent samples (or when appropriate Mann- Whitney U-test). A p-value of less than 0.05 was considered statistically significant. Analyses were performed using commercial software (IBM SPSS Statistics 20, SPSS Inc. An IBM Corp. Armonk, NY).

## RESULTS

**Fig.1:**
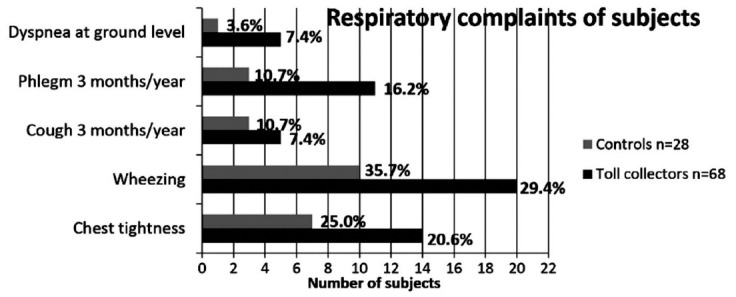
Respiratory complaints in subjects.

Forty toll collectors n(58.8%) and 16 controls n(57.1%) were active smokers, and no significant difference was observed between groups regarding smoking status (p=0.656) of the subjects are given in Fig.1.

Dyspnea at ground level (7.4% vs 3.6%) and phlegm in 3 months a year (16.2% vs 10.7%) in toll collectors were both higher than those in controls. No significant difference was observed regarding symptoms in toll collectors and controls (p>0.05). Spirometric values are shown in Table-I.

**Table-I:** Spirometric values of subjects.

	*Toll collectors n=68*	*Controls n=28*	*p-value*
FVC % predicted	105.5 ± 16.3	100.0 ± 9.5	0.101
FEV_1_% predicted	105.8 ± 15.7	103.1 ± 12.2	0.430
FEV_1_/FVC % predicted	83.6 ± 7.2	85.9 ± 7.0	0.168
MMFR % predicted	94.4 ± 27.9	98.1 ± 26.3	0.561
PEFR % predicted	100.9 ± 22.4	101.9 ± 21.4	0.850

There were no significant differences between groups when comparing the mean levels of % predicted spirometric values. The number of subjects with diurnal PEF variability and various complaints in both working and off working periods is shown in Table-II.

**Table-II:** Diurnal PEF variability in both working and off working periods.

	*Toll collectors*		*Controls*		*p-value*

	*n*	*%*	*n*	*%*	
PEF diurnal variability greater than 20% /working days	12	17.6	1	3.6	0.058
PEF diurnal variability greater than 20% /work off days	8	11.8	2	7.1	0.396

The number of toll collectors with diurnal PEF variability in working period was higher than that of the controls (p=0.058). The mean ECP level of TC was higher than that of controls, but the difference was not considered significant (p=0.088).

The mean ECP level of subjects with diurnal PEF variability was higher than that of subjects with no diurnal PEF variability, although the difference was not significant (p=0.41). Mean ECP levels related to the presence of various respiratory complaints and diurnal PEF variability during work and off-work periods are seen in Table-III.

**Table-III:** Mean ECP levels related to various complaints.

	*Mean ECP levels*	*p-value*
PEF Diurnal variability/work		
Yes n=25	29.1 ± 30.8	0.950
No n=71	29.6 ± 29.4	
Diurnal variability/off work		
Yes n=22	30.6 ± 32.4	0.842
No n=74	29.1 ± 28.9	
Smoking		
Yes n=56	29.6 ± 26.7	0.941
No n=21	30.8 ± 32.5	
Ex-smoker n=19	27.5 ± 35.6	
Wheezing		
Yes n=30	38.1 ± 39.6	0.052
No n=66	25.5 ± 23.0	
Chest tightness		
Yes n=21	27.3 ± 27.3	0.710
No n=75	30.1 ± 30.4	
Cough 3 months/year		
Yes n=8	19.5 ± 12.7	0.323
No n=88	30.3 ± 30.6	
Phlegm 3 months/year		
Yes n=14	26.8 ± 30.2	0.722
No n=82	29.9 ± 29.7	
Dyspnea at ground level		
Yes n=6	23.0 ± 14.3	0.583
No n=90	29.9 ± 30.3	

Mean ECP levels were higher in subjects with wheezing. Differences did not reach the level of statistical significance. Mean ECP levels negatively correlated with FEV_1_/FVC (r=-.095, p=0.359) and PEF (r=-.079, p=0.447), but correlations did not reach the level of statistical significance.

## DISCUSSION

The present study has shown higher rates of diurnal PEF variability during working periods and in serum ECP levels among toll collectors, although the differences between toll collectors and controls were not significant. The results of the present study have led the authors to consider the relationship between occupational DE exposure and asthmatic responses.

Present study did not detect differences between toll collectors and controls regarding to the prevalences of respiratory symptoms and pulmonary functions. In contrast to our study, there are some studies found the detrimental effects of DE on the respiratory system including complaints and lung functions.^17^,^18^

Few studies were comparable to the present study. DeToni et al. in a study including 290 traffic policemen, reported no difference in the 5-year follow-up data of FEV1 and FVC between traffic policemen and administrative workers.^19^

Arbak et al. found that the follow up of FEV1 and FVC values for four years did not show an accelerated decline in toll collectors compared to office workers.^20^ It can be postulated that, to be exposed to DE outdoors, such as seen among traffic policemen and toll collectors, might cause slow impairment in respiratory health.

One of the few studies using PEF in DE exposed humans showed that short-term exposure to DE at 300 μg/m3 caused irritation in upper airways, along with a temporary decline in PEF in healthy subjects, after only 75 min into exposure.^21^ The number of toll collectors with diurnal PEF variability in working period was higher than that of the controls in the present study. Moreover, the rate for diurnal PEF variability during off-work periods was higher among toll collectors.

ECP is one of the major cationic granule proteins released by activated eosinophils. It has a cytotoxic capacity against various microorganisms, as well as respiratory epithelial cells. ECP also stimulates mucous production in airways and histamine release by basophils and mast cells *in vitro*.^22^ Serum ECP has been found to directly correlate with activated eosinophils and to be a more sensitive marker of asthma severity than peripheral blood eosinophil counts in acute exacerbations.^23^ Although there have been a number of studies on ECP, it was not found to be a useful marker for asthma due to its lack of specificity.

Among TC with diurnal PEF variability in working periods, ECP levels were also high. The present study has some limitations including the lack of female subjects, measurement of indoor (manual toll booths) and outdoor levels of gaseous and particle components of DE. Technical deficiency for measuring both the gaseous and particle components of DE was another failure of this study.

## CONCLUSIONS

Serial PEF measurements combined with serum ECP measurements did not add a new tool to detect the sensitivity of workers dealing with DE. Much more diesel exhaust exposed workers should be included to search for cheap and available methods when evaluating airways.
